# The NF-κB Inhibitor, IMD-0354, Affects Immune Gene Expression, Bacterial Microbiota and *Trypanosoma cruzi* Infection in *Rhodnius prolixus* Midgut

**DOI:** 10.3389/fphys.2018.01189

**Published:** 2018-08-31

**Authors:** Cecilia S. Vieira, Otacílio C. Moreira, Kate K. S. Batista, Norman A. Ratcliffe, Daniele P. Castro, Patrícia Azambuja

**Affiliations:** ^1^Laboratório de Bioquímica e Fisiologia de Insetos, Instituto Oswaldo Cruz, Fundação Oswaldo Cruz (IOC/FIOCRUZ), Rio de Janeiro, Brazil; ^2^Laboratório de Biologia Molecular e Doenças Endêmicas, Instituto Oswaldo Cruz, Fundação Oswaldo Cruz (IOC/FIOCRUZ), Rio de Janeiro, Brazil; ^3^Laboratório de Biologia de Insetos, Universidade Federal Fluminense, Niterói, Brazil; ^4^College of Science, Swansea University, Wales, United Kingdom; ^5^Departamento de Entomologia Molecular, Instituto Nacional de Entomologia Molecular (INCT-EM), Rio de Janeiro, Brazil

**Keywords:** insect immune system, *Rhodnius prolixus*, antimicrobial peptides, Nf-κB transcription factor, *Trypanosoma cruzi*, microbiota

## Abstract

*Rhodnius prolixus* is an insect vector of *Trypanosoma cruzi*, the causative agent of Chagas disease in Latin America. Nuclear factor-*κ*B (NF-*κ*B) transcription factors (TF) are conserved components of the innate immune system in several multicellular organisms including insects. The drug IMD-0354 [*N*-(3,5-bis-trifluoromethyl-phenyl)-5-chloro-2-hydroxy-benzamide] is a selective inhibitor of I*κ*B kinases. It blocks I*κ*Bα phosphorylation thus preventing nuclear translocation of the NF-*κ*b TF. In humans, NF-*κ*B is involved in several biological processes such as inflammation, cell proliferation and immunity. In insects, the activation of the immune system upon microbial challenge can be controlled by signaling pathways such as the immune deficiency (IMD) and Toll, to combat infection. These activated pathways signal to downstream NF-*κ*B TF to stimulate specific immune genes, triggering the synthesis of several molecules such as the antimicrobial peptides. In *Drosophila melanogaster*, the activation and regulation of NF-*κ*B TF have been elucidated, while in triatomines these mechanisms are not fully understood Therefore, the present study investigated the effects of oral administration of the drug IMD-0354 on the *R. prolixus* immune response to challenge with bacteria and *T. cruzi*, as well as the impact on the gut bacterial microbiota. *R. prolixus* were fed with rabbit blood containing IMD-0354 and *Escherichia coli*, *Staphylococcus aureus*, or *T. cruzi*. The effects of IMD-0354 on insect mortality and antimicrobial activity in insect midgut samples, as well as the relative expression of *R. prolixus* immune genes were recorded. The bacterial microbiota was analyzed, and viable parasites were counted in insect midgut samples. The IMD-0354 treatment modulated antibacterial activity and the gene expression patterns of defensin A, defensin B, defensin C, and prolixicin, and the genes involved in the IMD and Toll pathways. Additionally, there was an increase of bacterial microbiota in treated insects. Insects treated with IMD-0354 and concomitantly infected with bacteria or *T. cruzi* through the blood meal had increased mortality, while the *T. cruzi* population in *R. prolixus* midgut was reduced. The inhibitory effect of IMD-0354 indicates the importance of NF-*κ*B TF in the innate immune responses involved in the control of bacteria and parasite infections in the *R. prolixus* midgut.

## Introduction

The invertebrate immune system relies on innate responses and lacks the classical adaptive immunity observed in vertebrates (Hoffmann and Reichhart, 2002). However, innate immunity is very effective as the first-line of host defense against infection in all eukaryotic organisms (Fearon and Locksley, 1996; Hoffmann and Reichhart, 2002). Nuclear Factor-Kappa B (NF-*κ*B) transcription factors (TF) are important and conserved multi-components from pathways that regulate the expression of many genes including those responsible for the innate immune system (Silverman and Maniatis, 2001; Li and Verma, 2002; Kumar et al., 2004). NF-*κ*B proteins have been identified in a range of multicellular organisms including cnidarians (Sullivan et al., 2007; Sinkovics, 2017), insects (Hetru and Hoffmann, 2009; Ganesan et al., 2011; Dong et al., 2012; Mesquita et al., 2015) and humans (Hayden and Ghosh, 2004; Senegas et al., 2015). Thus, these pathways emerged early in evolution, more than 500 million years ago, so that and the basic mechanisms of recognition and activation of response against pathogens are conserved in the animal kingdom (Hoffmann et al., 1999; Palmer and Jiggins, 2015).

In naïve animal cells, NF-*κ*B TF are usually associated with inhibitory IκB proteins (such as cactus protein in insects) which retain the TF in the cytoplasm (Belvin et al., 1995; Silverman and Maniatis, 2001; Häcker and Karin, 2006). In insects, the recognition of an invading microorganism activates immune signaling pathways, such as the IMD and Toll pathways, inducing the phosphorylation and cleavage of the I*κ*B inhibitory proteins (Li and Verma, 2002; Häcker and Karin, 2006) allowing NF-*κ*B TF (relish, dorsal) to translocate to the nucleus, triggering the transcription of several effector molecules such as the antimicrobial peptides (AMPs) (Ferrandon et al., 2007; Hetru and Hoffmann, 2009) to combat infection (Häcker and Karin, 2006; Lemaitre and Hoffmann, 2007). Additionally, *κ*B motifs have been detected in promoter regions of several AMPs genes from various insects such as *Drosophila melanogaster* (Kappler et al., 1993; Ganesan et al., 2011), *Hyalophora cecropia* (Engström et al., 1993), *Glossina morsitans* (Wang et al., 2008), and *Anopheles gambiae* (Christophides et al., 2002; Dong et al., 2012).

The drug IMD-0354 [*N*-(3,5-bis-trifluoromethyl-phenyl)-5-chloro-2-hydroxy-benzamide] is a selective molecular inhibitor of I*κ*K-2 (Tanaka et al., 2005; Sugita et al., 2009). It blocks I*κ*Bα phosphorylation thus preventing nuclear translocation of the transcription factor NF-*κ*B (Uota et al., 2012; Kong et al., 2015). *Rhodnius prolixus* is a triatomine species of medical importance vectoring *Trypanosoma cruzi*, the causative agent of Chagas disease in Latin America (Chagas, 1909; Vallejo et al., 2009; Coura, 2015; World Health Organization WHO, 2017). Therefore, studying aspects of *R. prolixus* immune system is relevant, for providing potential targets for insect control. In *D. melanogaster*, although the processes of activation and regulation of NF-*κ*B TF are well-known (Ferrandon et al., 2007; Ganesan et al., 2011; Buchon et al., 2014; Imler, 2014), in triatomines these pathways are not fully understood. For example, the gene encoding the protein Relish, a conserved TF related to IMD signaling pathway (Ferrandon et al., 2007), was identified in *R. prolixus* as *RpRelish*, but its inhibitory protein Caspar was not detected (Mesquita et al., 2015). In addition, Dorsal, a NF-*κ*B TF involved in the Toll pathway and its inhibitory protein, Cactus, a member of I*κ*B-family protein (Geisler et al., 1992; Ferrandon et al., 2007) were detected in *R. prolixus* (Ursic-Bedoya et al., 2009; Ribeiro et al., 2014). Regarding effector molecules regulated by immune signaling pathways, AMPs have been isolated from *R. prolixus* midgut and fat body, including defensins (Lopez et al., 2003), lysozymes (Ursic-Bedoya et al., 2008) and prolixicin (Ursic-Bedoya et al., 2011). The induction of these effector molecules has been studied in *R. prolixus* challenged with different microorganisms. AMPs are differentially regulated, depending upon whether infection occurs with Gram-positive or Gram-negative bacteria or protozoans (Vieira et al., 2014, 2015, 2016).

Therefore, the main objective of the present work was to determine the effect of IMD-0354 in the regulation of genes involved in NF-*κ*B pathways (relish, dorsal and cactus, respectively) and effector AMPs (defensins and prolixicin) in *R. prolixus* challenged with *T. cruzi* or bacteria. The results indicate that *R. prolixus* NF-*κ*B TF have a pivotal role in the modulation of the innate immune system and in the ability of triatomines to deal with different infections.

## Materials and Methods

### Bacterial Maintenance

*Staphylococcus aureus* 9518 and *Escherichia coli* K12 4401, both obtained from the National Collections of Industrial and Marine Bacteria (NCIMB), Aberdeen, United Kingdom, and *Serratia marcescens* RPH, previously isolated from *R. prolixus* midgut (Azambuja et al., 2004) were used in all experiments. Bacteria were maintained frozen at −70°C in brain heart infusion (BHI) plus 10% glycerol until use. For all experimental procedures, bacteria were cultivated with shaking (90 revolutions per minute) in 20 mL of tryptone soy broth (TSB) for 17 h at 30°C, and then 10 mL of fresh TSB were inoculated with 100 μL of the respective bacterial culture and incubated for a further 4 h under the same conditions. The bacteria were then washed in phosphate buffered saline (PBS, 0.01 M phosphate buffer, 0.0027 M potassium chloride and 0.137 M sodium chloride, pH 7.4) and diluted in TSB for use.

### *Trypanosoma cruzi* Culture

*Trypanosoma cruzi* Dm28c clone (COLPROT 0010) were provided from the Coleção de Protozoários da Fundação Oswaldo Cruz, Rio de Janeiro, Brazil (Fiocruz, COLPROT)^1^. Epimastigotes were cultivated at 28°C in modified brain heart infusion (BHI) media (Sigma–Aldrich), containing hemin and supplemented with 10% heat-inactivated bovine fetal serum (Azambuja and Garcia, 1997).

### Insect Feeding, Infection, and Treatment *in vivo*

*The R. prolixus* colony was maintained at *Laboratório de Bioquímica e Fisiologia de Insetos*, IOC/FIOCRUZ, at a relative humidity of 50–60% and at 27°C. For the experiments, *R. prolixus* 5th instar nymphs were randomly chosen and then fed with defibrinated rabbit blood through a membrane feeding device (Azambuja and Garcia, 1997). IMD-0354 (Sigma–Aldrich) was diluted in dimethyl sulfoxide (DMSO) (20 mg/mL) as a stock solution, according to the manufacturer’s instructions. For assaying the effects of IMD-0354 on bacterial and parasite infections insects were fed with defibrinated rabbit blood containing 5 or 10 μg of IMD-0354/mL. Additionally, the insects treated with IMD-0354 were concomitantly orally infected with bacteria, *E. coli* or *S. aureus* at a final concentration of 10^4^ bacteria/mL of blood or with *T. cruzi* epimastigotes at 1 × 10^6^ parasites/mL of blood (Vieira et al., 2014). IMD-0354 untreated control groups, infected or not, were fed on blood containing the same final concentration of the solvent DMSO (0,004%) as the treated groups. Parasite quantification (detailed below) was assessed in a Neubauer chamber under an optical microscope. Insects not fully engorged were discarded.

### Antibacterial Assays *in vitro*

Anterior midgut samples were collected from 5th instar nymphs of *R. prolixus* 7 days after feeding (DAF) on blood (control untreated group) or blood plus 5 μg of IMD-0354/mL (Sigma–Aldrich). From each group, 10 insect midguts were individually collected in 1.5 mL reaction tubes and diluted in 200 μL ultrapure water. The samples were homogenized, centrifuged at 10,000 ×*g* for 10 min at 4°C and the supernatants filtered using Millipore PVDF (0.22 μm) and maintained at −20 °C until use (Vieira et al., 2014, 2016). Midgut samples from control and treated insects were incubated for 19 h at 37°C with *E. coli*, *S. aureus* or *S. marcescens* (Vieira et al., 2014). The midgut antibacterial activity was measured by the turbidimetric assays (TB) using a Spectra Max 190 Plate Reader (Molecular Devices, Sunnyvale, CA, United States), as described previously (Castro et al., 2012; Vieira et al., 2016).

### RT-qPCR Assays

Temporal relative expression of *R. prolixus* antimicrobial peptides genes, defensins and prolixicin (*defA, defB, defC, prol*) and Toll and IMD pathway related genes (*RpDorsal, RpCactus*, and *RpRelish*) were analyzed through reverse transcription quantitative PCR. The specific primers for the genes of interest, as well as *R. prolixus* housekeeping genes (*α-tubulin* and *GAPDH*), were designed and used as previously published: *α-tubulin* and *GAPDH* (Paim et al., 2012), *defA, defB* and *defC* (Lopez et al., 2003; Vieira et al., 2016); *prol* (Ursic-Bedoya et al., 2011; Vieira et al., 2016), *RpCactus* (Ribeiro et al., 2014), *RpRelish, RpDorsal* (Mesquita et al., 2015) (**Supplementary Material**). The relative quantification of gene expression was estimated using untreated insects (controls) as calibrators. Untreated control and IMD-0354-treated *R. prolixus* 5th instar nymphs were dissected at different days after feeding to collect and separate midgut samples in three pools containing five anterior midguts each (Vieira et al., 2016). Total RNA was extracted and quantified using a NucleoSpin^®^ RNA II Kit (Macherey-Nagel, Düren, Germany) and a NanoDrop 2000 Spectrophotometer (Thermo Scientific, Waltham, MA, United States), respectively. Synthesis of cDNA was with a First-Strand cDNA Synthesis Kit (GE Healthcare, Buckinghamshire, United Kingdom) using 2.5 μg of total RNA and the pd(N)6 primer. Quantification of cDNA was assessed by fluorescence in a Qubit Fluorimeter (Life Technologies) with the ssDNA assay kit. Real-time quantitative polymerase chain reactions (RT-qPCR) were conducted in an ABI PRISM 7500 Sequence Detection System (Applied Biosystems) at the FIOCRUZ facilities (Real-Time PCR Platform RPT-09A) using GoTaq^®^ qPCR Master Mix (PROMEGA). Gene expression assays and analyses were performed as previously described by Vieira et al. (2016).

### Microbiota Analysis

Total midguts from *R. prolixus* 5th instar nymphs were collected 7 days after feeding with blood with or without IMD-0354. It was performed three independent experiments with 10 insects from each group (*n* = 30). Cultivable bacteria microbiota population were quantified using the colony forming units (CFU) procedure. The samples were serially tenfold diluted with sterile PBS in 1.5 mL reaction tubes. Then 20 μL of each dilution was spread on BHI agar (Sigma–Aldrich) plates and immediately incubated at 30°C for 24 h and the CFU counted. Autoclaved PBS was also plated and incubated to assess the sterility of all experiments. Additionally, RT-qPCR was performed to analyze relative expression of 16S-rRNA from *S. marcescens*, *Rhodococcus rhodnii* and Enterococcaceae using cDNA from 3 pools of 10 anterior midguts from insects: untreated; treated with IMD-0354; infected with *T. cruzi* epimastigotes; treated with IMD-0354 simultaneously infected with *T. cruzi*e pimastigotes.

### Analysis of IMD-0354 Effects on *Rhodnius prolixus* Mortality

To determine the toxicity of IMD-0354 in *R. prolixus* 5th instar nymphs, mortalities were evaluated 10 days after feeding. The groups analyzed were: control (fed only on blood), insects fed on blood containing 5 μg IMD-0354/mL, insects fed on blood containing 5 μg IMD-0354/mL plus *T. cruzi* Dm28c or *E. coli* or *S. aureus.* Each blood meal type Was replicated twice, and 30 insects Were used for each replicate.

### Quantification of Parasites in the Digestive Tract

Fifth instar *R. prolixus* nymphs fed With *T. cruzi* epimastigotes Were dissected and the total digestive tract Was collected 20 DAF in 1.5 mL microtubes and homogenized in 1.0 mL of sterile PBS. Parasite number Was determined by counting in a Neubauer haemocytometer and expressed as parasites/mL. Parasites quantification Was performed in three independent experiments With five individually insects each (n = 15).

### Statistical Analysis

The statistical analysis of data was performed using one-way ANOVA, two-way ANOVA, Student’s T, Tukey’s or Mann–Whitney tests on GraphPad Prism 5 software, depending on data distribution. Significant differences between groups are displayed in each figure and legends in the present study and were considered statistically different when *p* < 0.05.

### Ethics Declaration

The colony of *R. prolixus* was maintained in controlled environmental conditions at *Laboratório de Bioquímica e Fisiologia de Insetos* of IOC-FIOCRUZ and fed with defibrinated rabbit blood obtained at the *Instituto de Ciência e Tecnologia em Biomodelos* (ICTB), according to the Ethical Principles in Animal Experimentation approved by the *Comitê de Ética de Experimentação Animal* (CEUA/FIOCRUZ, under the protocol number LW019/17). The protocol was developed by CONCEA/MCT^2^, which is associated with the American Association for Animal Science (AAAS), the Federation of European Laboratory Animal Science Associations (FELASA), the International Council for Animal Science (ICLAS) and the Association for Assessment and Accreditation of Laboratory Animal Care International (AAALAC).

## Results

### Preliminary Observations

To evaluate IMD-0454 toxicity, initial experiments, tested feeding 5 or 10 μg IMD-0354/mL blood to insects. At the 10 μg level, *R. prolixus* mortality rate was greater than 60%, a fact that impaired the infection experiments (data not shown). So, at 5 μg IMD-0354/mL a similar mortality rate (15%) occurred as in the control insects (12%) (data not shown) then, that dosage was used in all subsequent experiments. Additionally, at 5 μg IMD-0354/mL had no direct effect on parasite and bacteria cultures after 24 h of incubation.

### IMD-0354 Affects the Expression of Genes Related to *R. prolixus* Immunity in the Midgut

**FIGURE 1:**
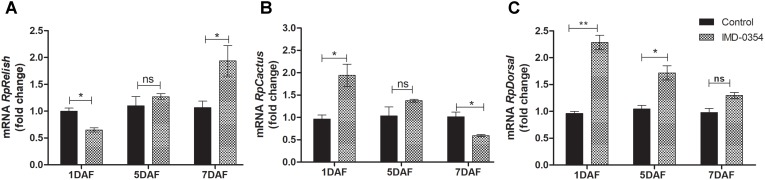
IMD-0354 effects on the relative gene expression of Relish, Cactus and Dorsal in the anterior midgut of *Rhodnius prolixus*. Relish, Cactus and Dorsal gene expressions were analyzed using the anterior midgut of fifth instar *R. prolixus* nymphs at different days after feeding on blood containing IMD-0354 (5 μg/ml). Data were quantified using the gene expression of untreated control insects as the calibrator (black columns) and shown as the relative expression of **(A)** *RpRelish*, **(B)** *RpCactus*, **(C)** *RpDorsal* on the 1st, 5th, and 7th days after feeding (DAF). Bars represent the mean ± SEM of 3 independent experiments with 3 pools of insects (*n* = 3). Means were compared using Student’s *T*-test; ^∗^*p* < 0.05, ^∗∗^*p* < 0.01, ns, non-significant.

**FIGURE 2:**
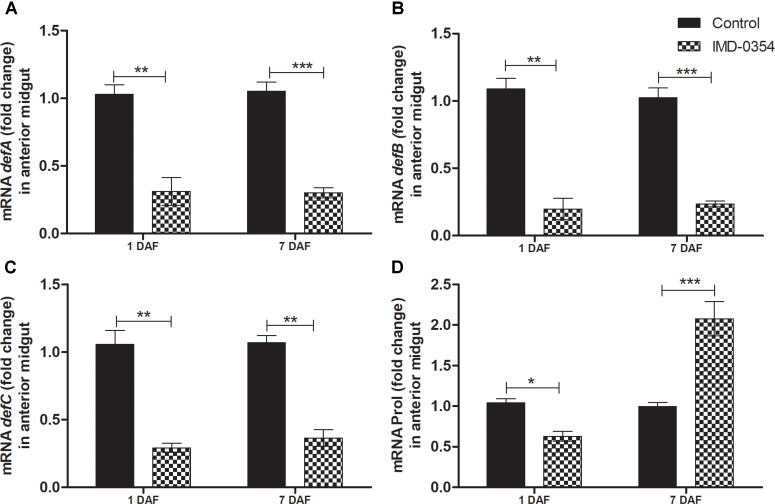
IMD-0354 effects on the relative gene expression of antimicrobial peptides in the anterior midgut of *Rhodnius prolixus.* Fifth instar nymphs of *R. prolixus* were fed on blood containing IMD-0354 (5 μg/ml). Data were quantified using the gene expression of untreated control insects as the calibrator (black columns). The grid columns show the relative expression of the antimicrobial peptide genes at the 1st and 7th days after feeding with IMD-0354. Relative expression of: **(A)** *defA*; **(B)** *defB* **(C)** *defC* **(D)** *Prol* at the 1st and 7th days after feeding. Treatments: black columns – insects fed with blood (control); grid columns – insects fed with blood containing the drug IMD-0354. Each bar represents 3 independent experiments performed in duplicate (*n* = 6). Means were compared using one-way ANOVA and Student *T*-test; ^∗∗∗^*p* < 0.001, ^∗∗^*p* < 0.01, ^∗^*p* < 0.1.

To test if oral administration of the drug IMD-0354 (5ug/mL) alters the regulation of the NF-*κ*B TF genes in the *R. prolixus* midgut, quantification of the temporal gene expression of TF *RpRelish, RpDorsal* and the inhibitory TF gene *RpCactus*, as well as some AMPs, was performed by RT-qPCR. The IMD-0354 treatment induced a significant downregulation of TF *RpRelish* gene expression and an upregulation of *RpCactus* and *RpDorsal* 1 DAF in comparison to control insects (**Figure 1A**, *p* < 0.05; **Figure 1B**, *p* < 0.05; and **Figure 1C**, *p* < 0.01). At 5 DAF, however, both *RpRelish* and *Rp*Cactus expressions are similar to untreated control insects while *Dorsal* expression is higher in treated insects (*p* < 0.05). In contrast, at 7 DAF, *RpRelish* levels were significantly higher, *RpCactus* mRNA levels were significantly lower than control insects (**Figures 1A,B**, *p* < 0.05). *RpDorsal* levels in treated or untreated insects are the same at 7 DAF (**Figure 1C**). The IMD-0354 treatment induced a significantly downregulation of the *defA*, *defB* and *defC* genes in the anterior midgut compared with control insects at 1 and 7 DAF (**Figure 2A**, *p* < 0.001, *p* < 0.01; **Figure 2B**, *p* < 0.001, *p* < 0.001; and **Figure 2C**, *p* < 0.01, *p* < 0.01). *Prol* expression was also lower at 1DAF in treated insects in comparison to control insects 1 DAF (**Figure 2D**, *p* < 0.01) but, in contrast, at 7 DAF, prol was higher than the control group (**Figure 2D**, *p* < 0.001).

### IMD-0354 Affects Antimicrobial Peptides Activity in *R. prolixus* Midgut

**FIGURE 3:**
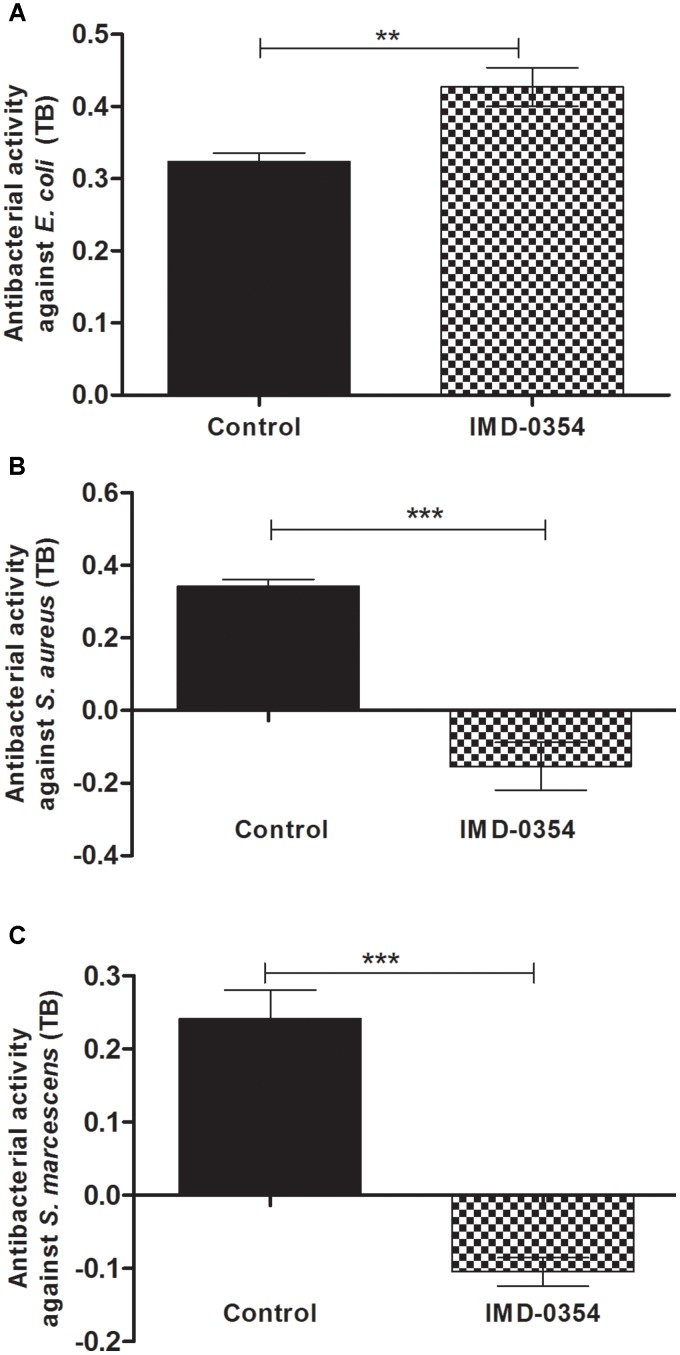
IMD-0354 effects on antibacterial activity in the anterior midgut of *Rhodnius prolixus.* Fifth instar nymphs of *R. prolixus* were fed on blood containing IMD-0354 (5 μg/ml). The antibacterial activities were measured *in vitro* using the anterior midgut samples 7 days after feeding and tested against **(A)** *E. coli*, **(B)** *S. aureus*, and **(C)** *S. marcescens* through the turbidimetric assay (OD_550_ nm) after 19 h incubation. Black column – untreated control insects fed only on blood; grid column – insects fed on blood containing IMD-0354. Bars represent the mean ± SEM of three independent experiments with nine pools of insects (*n* = 9). Means were compared using one-way ANOVA and Student’s *T*-test; ^∗∗∗^*p* < 0.001, ^∗∗^*p* < 0.01, NS, not significant.

The antibacterial assays performed with midgut samples from insects fed on blood with or without IMD-0354 was an attempt to corroborate AMP activity and gene expression. In fact, the IMD-0354 treatment modulated both AMP gene expression and antibacterial activity in the *R. prolixus* midgut (**Figure 3**). Anterior midgut samples from treated insects showed significantly lower activity against *S. aureus* and *S. marcescens* in comparison to the untreated control (**Figure 3B**, *p* < 0.001, **Figure 3C**, *p* < 0.001). In contrast, a higher antibacterial activity against *E. coli in vitro* was detected in treated insects compared to the controls (**Figure 3A**, *p* < 0.01).

### *R. prolixus* Midgut Bacterial Population Was Altered After IMD-0354 Treatment

**FIGURE 4:**
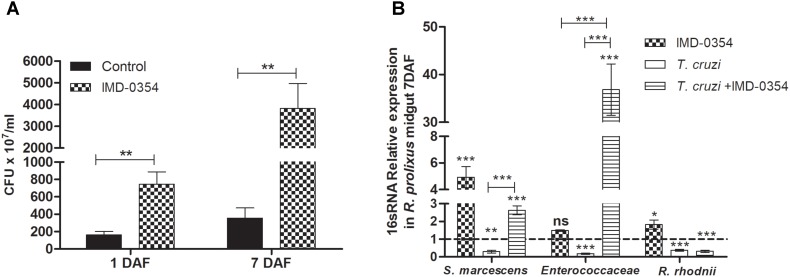
IMD-0354 effects on microbiota population in *Rhodnius prolixus* anterior midgut. Determination of bacterial load in the midgut of 5th instar nymphs of *R. prolixus* fed with blood containing IMD-0354 (5 μg/ml). **(A)** Colony forming units (CFU) were counted in the anterior midgut samples 1 and 7 days after feeding. **(B)** Relative expression of 16S-rRNA of *Serratia marcescens*, Enterococcaceae and *Rhodococcus rhodnii* analyzed by RT-qPCR. Data was normalized to the *R. prolixus* 18S RNA gene and quantified using the gene expression of untreated control insects as the calibrator represented by dotted lines on each graph. Black column – untreated control insects fed on blood; grid column – insects fed on blood containing IMD-0354; white column – insects fed on blood containing *T. cruzi* epimastigotes (10^6^ parasites/mL of blood); striped columns - insects fed on blood containing IMD-0354 plus *T. cruzi* epimastigotes (10^6^ parasites/mL of blood). Bars represent the mean ± SEM of three independent experiments with 10 insects (*n* = 30). Means were compared using Student’s *T*-test or Mann–Whitney test; ^∗∗∗^*p* < 0.001, ^∗∗^*p* < 0.01, ^∗^*p* < 0.05.

To investigate whether the treatment with a NF-*κ*B TF inhibitor could impact natural midgut homeostasis, the cultivable bacterial population was analyzed in the *R. prolixus* midgut at different times after drug ingestion. Insects fed with blood containing IMD-0354 had higher CFU counts than control insects at 1 and 7 DAF (*p* < 0.01) (**Figure 4A**). Furthermore, analysis of 16S RNA confirmed a significant increase of *S. marcescens* and *R. rhodnii* population in insects treated with IMD-0354 comparing to control insects (*p* < 0.001, *p* < 0.05) (**Figure 4B**). Additionally, in insects treated with IMD-0354 and concomitantly infected with *T*. *cruzi*, the population of *S. marcescens* and Enterococcaceae were significantly higher than untreated *T. cruzi* infected insects (*p* < 0.001, *p* < 0.001) (**Figure 4B**).

### IMD-0354 Treatment Compromises *R. prolixus* Survival After Microbial Challenge

**FIGURE 5:**
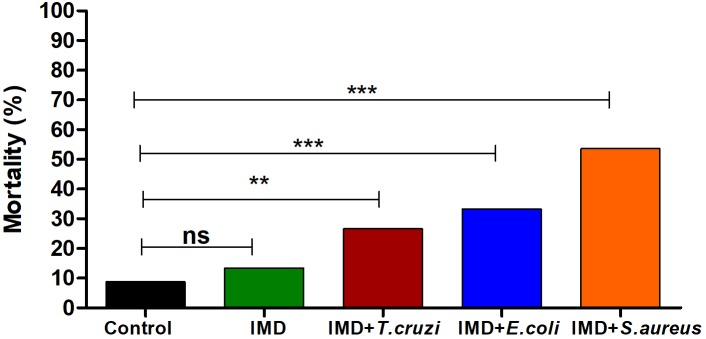
IMD-0354 effects on the mortality of *Rhodnius prolixus* infected with bacteria or *Trypanosoma cruzi*. *R. prolixus* 5th instar nymphs were fed on blood containing IMD-0354 (5 μg/ml) and *E. coli* or *S. aureus* at a final concentration of 1x 10^4^ bacteria/ml or *T. cruzi* at 1 × 10^6^ epimastigotes/ml. Numbers of dead insects were counted 7 days after the blood meal. Treatments: black column – untreated control insects fed on blood; green column - insect feed on blood with –IMD-0354; red column –IMD-0354 plus *T. cruzi*; blue column –IMD-0354 plus *Escherichia coli*.; orange column - IMD-0354 plus *Staphylococcus aureus.* Bars represent the percentage of dead insects (among 30 insects in each treatment) in 3 independent experiments. Means were compared using one-way ANOVA and Tukey’s post-test; ns ^∗∗^*p* < 0.01, ^∗∗∗^*p* < 0.001, ns, not significant.

The importance of NF-*κ*B TF in *R. prolixus* survival after a microbial challenge was analyzed by recording mortalities 72 h after a blood meal. Adding *T. cruzi* or bacteria to the blood of IMD-0354 treated insects increased the mortality to 31, 42, and 59% after *T. cruzi*, *E. coli*, and *S. aureus* oral infection, respectively (**Figure 5**). Insects infected with *E. coli*, *S. aureus* or *T. cruzi* without IMD-0354 treatment, had a similar mortality rate to control insects, lower than 18% (data not shown).

### Quantification of *T. cruzi* in the *R. prolixus* Treated With IMD-0354

**FIGURE 6:**
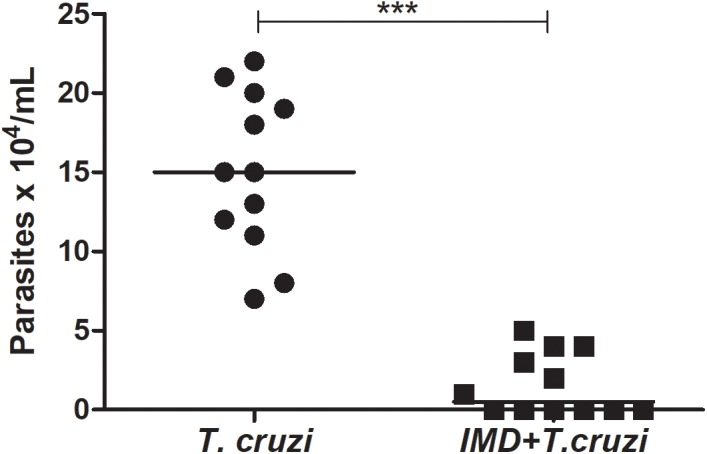
IMD-0354 effect on *Trypanosoma cruzi* population in the digestive tract of *Rhodnius prolixus*. *R. prolixus* 5th instar nymphs were fed on blood containing IMD-0354 (5 μg/ml of blood) and *T. cruzi* epimastigotes at a final concentration of 10^6^ parasites/mL of blood. Number of *T. cruzi* were estimated in *R. prolixus* 5^th^ instar nymphs in whole digestive tracts at 20 days after feeding (DAF). Each point represents the number of parasites in an individual insect, and bars indicate the median. Means were compared using Student *T*-test; ^∗∗∗^*p* < 0.001.

To investigate the role of NF-*κ*B TF in the establishment of *T. cruzi* in *R. prolixus* gut, insects were concomitantly treated with IMD-0354 and infected with *T. cruzi* epimastigotes through the blood meal. The parasite population was quantified in the digestive tract collected from 15 insects 20 DAF. The *T. cruzi* population in the gut of insects treated with IMD-0354 was significantly lower in comparison to untreated infected insects (**Figure 6**, *p* < 0.001). Moreover, there was a decrease of 50% in the percentage of insects infected after IMD-0354 treatment.

### IMD-0354 Affects the Induction of *defC* and *prol* Genes After *T. cruzi* Infection in *R. prolixus* Midgut

**FIGURE 7:**
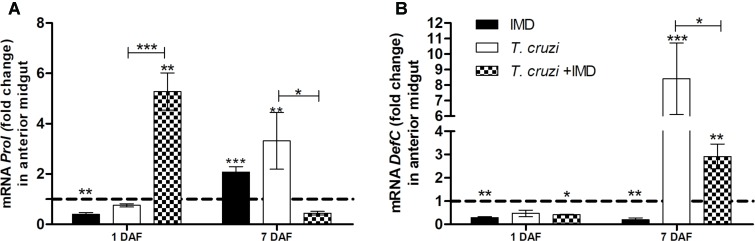
IMD-0354 effects on relative gene expression of antimicrobial peptides in the midgut of *Rhodnius prolixus* infected with *Trypanosoma cruzi*. *R. prolixus* 5th instar nymphs were fed on blood containing IMD-0354 and *T. cruzi* epimastigotes at a final concentration of 10^6^ parasites/mL. Data were quantified using the gene expression of untreated control insects as the calibrator represented by horizontal dotted lines on each graph to show the relative expression of antimicrobial peptide. **(A)** *Prolixicin* relative expression; **(B)** *Defensin C* relative expression on the 1st and 7th days after feeding. Treatments: Black column- insect feed on blood containing IMD-0354 alone; white columns – fed on *T. cruzi* alone; grid columns – insects fed on IMD-0354 and *T. cruzi*. Each bar represents 3 independent experiments performed in duplicate (*n* = 6). Means were compared using two-way ANOVA and Student *T*-test; ^∗∗∗^*p* < 0.001, ^∗∗^*p* < 0.01, ^∗^*p* < 0.05.

After observing that IMD-0354 impedes *T. cruzi* infection in *R. prolixus* midgut whether in these infected insects the drug altered the expression of *defC* and *prol* was tested. Defensin C and prolixicin genes were chosen for these experiments since previous results demonstrated the upregulation of these genes after *T. cruzi* infection (Vieira et al., 2016). Quantification of gene expression was examined at 1 and 7 DAF. At 1 DAF, *T. cruzi* infected insects concomitantly treated with IMD-0354 had a higher *prol* expression in comparison to controls and to *T. cruzi* infected untreated insects (**Figure 7A**, *p* < 0.01, *p* < 0.001, respectively). In contrast, on 7DAF, *prol* levels in the midgut of infected insects concomitantly treated with IMD-0354 were 8-fold lower in comparison to *T. cruzi* infected untreated insects (**Figure 7A**, p < 0.05). Unlike the *prol* gene expression, that of the *defC* gene at 1DAF showed no differences between infected insects and insects infected with *T. cruzi* and concomitantly treated with IMD-0354. However, on the 7 DAF, *defC* levels were lower in *T. cruzi* infected/IMD-0354 treated insects in comparison to infected but untreated insects (**Figure 7B**, *p* < 0.05). Both *prol* and *defC* genes were also significantly upregulated after *T. cruzi* infection in comparison to control insects 7DAF (**Figure 7A**, *p* < 0.01; **Figure 7B**, *p* < 0.001, respectively).

## Discussion

The innate immune system represents the first line of defense against infection by pathogens. For decades, insect innate immunity has commonly been studied using the genetic malleable model, *D. melanogaster.* This model elucidated details of the genes involved in the Toll and IMD pathways, as well as their specific roles in combating infection, by challenging with Gram-positive and negative bacteria and fungi (Lemaitre and Hoffmann, 2007; Ferrandon et al., 2007; Hetru and Hoffmann, 2009).

Nowadays, attention has also turned to the immune systems of insect vectors of disease in order to identify potential targets to develop new control strategies. Regarding the triatomine vector, *R. prolixus*, only recently its whole genome sequence was published (Mesquita et al., 2015). With these available data now, it is possible to study the pattern recognition mechanisms and genes involved in immune signaling pathways as well as the effector genes regulated by these pathways. In this context, in the present work, the compound IMD-0354 was used as an immunosuppressive drug, since it is selective NF-*κ*B TF inhibitor, preventing the transcription of several antimicrobial peptides in mammals (Kamon et al., 2004; Tanaka et al., 2005; Sugita et al., 2009). The use of this drug in arthropods, as far as we are aware, has not previously been documented to study components of insect vector immunity. Through drug incorporation in the insect blood meal, the importance of genes regulated by NF-*κ*B pathways in the activation and modulation of the *R. prolixus* immune genes against infection by different microorganisms can be evaluated, as well as the influence of Toll and IMD TF in the establishment of *T. cruzi* infections.

The lack of some canonical elements from the IMD pathway was observed in the *R. prolixus* genome, with the absence of some genes such as IMD, FADD (Fas-Associated protein with dead domain) and Caspar, the inhibitor of the IMD pathway (Mesquita et al., 2015). The gene encoding the TF Relish, however, was detected and its knockdown induced a decrease of some AMPs and allowed the proliferation of the intestinal microbiota in *R. prolixus* (Mesquita et al., 2015), indicating the presence of a functional IMD pathway. Some disparity in the IMD signaling pathway elements is also observed in other insects such as in *Pediculus humanus* (Kim et al., 2011), *Acyrthosiphon pisum* (Gerardo et al., 2010) and in the ticks, *Ixodes scapularis* (Shaw et al., 2017), *Rhipicephalus microplus* (Rosa et al., 2016) suggesting some plasticity in the IMD pathway among arthropods (Palmer and Jiggins, 2015; Shaw et al., 2017).

When *R. prolixus* 5th instar nymphs were fed with blood containing IMD-0354, *defA*, *defB*, and *defC* expression and activity were downregulated, suggesting that NF-*κ*B TF activation and its translocation to the nucleus is essential to trigger the transcription of, at least, the defensins genes in *R. prolixus*. In contrast, the NF-*κ*B inhibitor induced an increase in *prol* levels at 7 DAF. This discrepancy may be due to the fact that the *R. prolixus* defensins, and those of other triatomines such as *Triatoma brasiliensis* and *Panstrongylus megistus*, are regulated by NF-*κ*B TF (Lopez et al., 2003; Waniek et al., 2009; de Araújo et al., 2015), whereas NF-*κ*B binding sites are absent in the prolixicin gene sequence, which appears to be regulated by GATA transcription factors (Ursic-Bedoya et al., 2011). Further experiments are necessary to fully understand the signaling pathways that regulate prolixicin in *R. prolixus* and in other hemipterans. The increase of prolixicin mRNA levels in the *R. prolixus* midgut after NF-*κ*B inhibition by IMD-0354 could represent an indirect compensatory effect that implicates other pathways, such as those controlled by GATA TF, for regulating the genes.

The low antibacterial activity detected in the midgut samples of insects treated with IMD-0354, against the Gram-positive *S. aureus* and the Gram-negative *S. marcescens* might be related to the negative regulation of *defA*, *defB*, and *defC*. In contrast, the increased antibacterial activity against *E. coli* which could be related to the enhancement of the *prol* expression. Previously, in *in vitro* studies, of purified prolixicin from *R. prolixus* fat body have been shown to have strong activity against *E. coli* (Ursic-Bedoya et al., 2011). In addition, the increase of cultivable bacterial numbers, as well *S. marcescens*, *R. rhodnii* and Enterococcaceae in the midgut in IMD-0354-treated insects suggests microbiota regulation by the defensins, highlighting the importance of NF-*κ*B TF in *R. prolixus* intestinal homeostasis.

Moreover, knowledge of the role of immune signaling pathways and effector proteins (AMPs) in the regulation of insect intestinal bacterial microbiota is still fragmentary. Since AMP synthesis can inhibit the development of pathogens in their insect vectors (Vizioli et al., 2001a,b; Boulanger et al., 2002, 2004; Telleria et al., 2013; Das De et al., 2018), innumerable microorganisms present in the intestinal tract are able to induce the insect immune system (Cirimotich et al., 2011; Eappen et al., 2013; Vieira et al., 2014), consequently interfering in the life cycle of the parasites. *S. marcescens*, one of the most abundant bacteria found in *R. prolixus* gut (da Mota et al., 2012), possesses lytic mechanisms that drastically reduce the success of *T. cruzi* infection in the *R. prolixus* digestive tract (Azambuja et al., 2004; Castro et al., 2007, 2012; Vieira et al., 2016). It was suggested that the normal *S. marcescens* population growth is inhibited by *R. prolixus* AMPs, especially by defensins (Vieira et al., 2014, 2016). *T. cruzi* Dm28c infection in *R. prolixus* seems to overcome the trypanolytic effect of *S. marcescens*, inducing a strong upregulation of *defC* and *prol* (Vieira et al., 2015, 2016) which in turn decrease the *S. marcescens* population.

In the present study, IMD-0354 affected the normal signaling requirements needed for AMP transcription and protein synthesis since the drug prevents the translocation of the TF to the nucleus (Tanaka et al., 2005). A transitory modulation in transcriptional levels of NF-kB TFs (*RpRelish*, *RpDorsal*)and of IkB *RpCactus* was observed in IMD-0354 treated insects. Since insects treated withIMD-0354recordedan increased population of some bacterial species in the midgut, this effect might be associated with the activation of the Toll pathway, marked by an enhancementinmRNA levels of Cactus (IkB) and Dorsal(NF-kBTF). Theopposite occurs in IMD pathways, in which the TF, Relish, is downregulated. Several studies using different insect species have demonstrated the role of the gut microbiota in stimulating immune signaling pathways (Eappen et al., 2013; Eleftherianos et al., 2013; Jiménez-Cortés et al., 2018). After the recognition of bacterial components by specific receptors, the Toll pathway is activated, requiring the degradation of the Cactus protein to release Dorsal protein, allowing its nuclear translocation to initiate the transcription of effector genes (Ferrandon et al., 2007; Ganesan et al., 2011). These processes are followed by the transcription of both Cactus and Dorsal mRNAs, in order to rescue and retain these regulatory proteins in the cytoplasm (Geisler et al., 1992; Nicolas et al., 1998; Rutschmann et al., 2002). It is known that the drug IMD-0354 acts downstream in microbial recognition and upstream of NF-kB TF (Relish and Dorsal) nuclear translocation, only impairing the phosphorylation of IkB proteins (Cactus) but not the transcription of the genes (NF-kB TF and IkB) (Tanaka et al., 2005). Thus, the alterations in Relish, Cactus and Dorsal gene expressions detected here could represent secondary effects of IMD-0354 treatment in *R. prolixus*.

The main effect of IMD-0354 treatment is to inhibit AMPs transcription. In the present work, the three defensins studied were suppressed in IMD-0354 treated insects. Downregulation of the AMP genes confirmed by the lower antibacterial activity detected in *R. prolixus* midgut samples was one of the possible reasons that allowed an intense proliferation of *S. marcescens*, *R. rhodnii* and Enterococcaceae. Thereby, defensins and its related NF-*κ*B signaling pathways seem to play an essential role in regulating microbiota homeostasis in *R. prolixus* midgut. In this sense, the negative impact on the *T. cruzi* population was observed in insects fed with the NF-*κ*B inhibitor could be related to the increased bacterial population of *S. marcescens* and Enterococcaceae. As discussed above, *S. marcescens* presents trypanolytic activity (Azambuja et al., 2004; Castro et al., 2007) and large amounts of this bacteria in the midgut probably interfered in *T. cruzi* development in IMD-0453 treated insects. Additionally, a decrease in the *defC* and *prol* levels in insects fed with IMD-0354 concurrently infected with *T. cruzi*, reinforcing the importance of the activation of these genes for the establishment of *T. cruzi* infection in *R. prolixus*. Together these results highlight the relevance of genes regulated by NF-*κ*B TF in the *R. prolixus* immune system and gut homeostasis, as well as in the development of the parasite in the insect vector. Studying the genes involved in the signaling pathways regulated by NF-*κ*B TF is essential for a broad knowledge of the mechanisms of activation and modulation of the immune response. More experiments are necessary to understand how different microorganisms selectively activate immune signaling pathways and different NF-*κ*B TF molecules. Investigation of NF-*κ*B TF genes and genomic analysis of AMPs promoter regions could provide relevant insights into the function of each pathway in controlling effector molecules against specific invading microorganisms.

## Author Contributions

CSV conceived and designed the experiments, performed all the experiments, participated in the analysis and interpretation of the data, wrote the manuscript, and corrected and approved the final manuscript. OCM conceived and designed the PCR experiments, participated in the analysis and interpretation of the data, and read, corrected, and approved the final manuscript. KSB performed the experiments of feeding and infection in insects, parasite quantification in insect midgut, participated in the analysis and interpretation of the data, and read, corrected, and approved the final manuscript. NAR conceived and designed the experiments, wrote the manuscript, and corrected and approved the final manuscript. DPC participated in the physiological and molecular experiments, analysis and interpretation of the data, and read, corrected, and approved the final manuscript. PA conceived and designed the experiments, contributed with reagents and materials, participated in the analysis and interpretation of the data, and read, corrected, and approved the final manuscript.

## Conflict of Interest Statement

The authors declare that the research was conducted in the absence of any commercial or financial relationships that could be construed as a potential conflict of interest.

## Footnotes

1 http://colprot.fiocruz.br/

2 http://www.sbcal.org.br/
