# Supplementary material for: The NF-κB Inhibitor, IMD-0354, Affects Immune Gene Expression, Bacterial Microbiota and Trypanosoma cruzi Infection in Rhodnius prolixus Midgut
Source: Front Physiol. 2018 Aug 31;9:1189. doi: 10.3389/fphys.2018.01189 (PMC6128222; doi:10.3389/fphys.2018.01189)
Supplement: Supplementary file 1 [file Table_1.DOCX]

**Table**

**Additional file 1: Oligonucleotide primers used for qPCR analysis**

| Gene/name | Sequence 5’-3’ | Amplicon length | Reference |
| --- | --- | --- | --- |
| GAPDH-F | GATGGCGCCCAGTACATAGT |  |  |
| GAPDH-R | AGCTGACGGGGCTGTTATTA | 111 bp | Paim et al., 2012 |
| TUB-F | TTTCCTCGATCACTGCTTCC |  |  |
| TUB-R | CGGAAATAACTGGGGCATAA | 129 bp | Paim et al., 2012 |
| RPDEFA-F | GAATACTCCACTCAACCGCAAC |  |  |
| RPDEFA-R | agggcatcatctagttgttgatgagtg | 131 bp | Vieira et al., 2016 |
| RPDEFB-F | GGATATTCCACTCAACCGCAAC |  |  |
| RPDEFB-R | agagcatcgtctaattcttgttgagtg | 131 bp | Vieira et al., 2016 |
| RPDEFC-F | CAGTACAGTCCTAATACCTAGCC |  |  |
| RPDEFC-R | tgggcatcatctaattgatgttgagaa | 136 bp | Vieira et al., 2016 |
| F1 (prolixicin) | ACAATTTTGGTGGTGGTTGTC |  |  |
| qR (prolixicin) | GCTTGAGCTCTGGTCCTTCC | 194 bp | Ursic-Bedoya et al., 2011 |
| Rp Relish - R | GCAGCCCCAAAGTTCTTACA |  |  |
| Rp Relish-F | TTTTTCGTGAGCAACTGGTG | 76 bp | Mesquita et al., 2015 |
| Rp Cactus – R | GGAGTCGGACGATACCTCAA |  |  |
| Rp Cactus - F | GTGCTGGTGCTTGTACGAAA | 78 bp | Mesquita et al., 2015 |
| Rp Dorsal – R  Rp Dorsal - F | CAATGGTCGTTCTTGGACT  CAACAGCTGCTAAACCGACA | 77 bp | Mesquita et al., 2015 |
